# Protective Mechanisms of Flavonoids in Parkinson's Disease

**DOI:** 10.1155/2015/314560

**Published:** 2015-10-20

**Authors:** Kasthuri Bai Magalingam, Ammu Kutty Radhakrishnan, Nagaraja Haleagrahara

**Affiliations:** ^1^Department of Pathology, Faculty of Medicine and Health, International Medical University, Bukit Jalil, 57000 Kuala Lumpur, Malaysia; ^2^Discipline of Biomedicine, College of Public Health, Medical and Veterinary Sciences, James Cook University, Townsville, QLD 4811, Australia

## Abstract

Parkinson's disease is a chronic, debilitating neurodegenerative movement disorder characterized by progressive degeneration of dopaminergic neurons in the *substantia nigra pars compacta* region in human midbrain. To date, oxidative stress is the well accepted concept in the etiology and progression of Parkinson's disease. Hence, the therapeutic agent is targeted against suppressing and alleviating the oxidative stress-induced cellular damage. Within the past decades, an explosion of research discoveries has reported on the protective mechanisms of flavonoids, which are plant-based polyphenols, in the treatment of neurodegenerative disease using both *in vitro* and *in vivo* models. In this paper, we have reviewed the literature on the neuroprotective mechanisms of flavonoids in protecting the dopaminergic neurons hence reducing the symptoms of this movement disorder. The mechanism reviewed includes effect of flavonoids in activation of endogenous antioxidant enzymes, suppressing the lipid peroxidation, inhibition of inflammatory mediators, flavonoids as a mitochondrial target therapy, and modulation of gene expression in neuronal cells.

## 1. Introduction

James Parkinson (1817), in his paper entitled “Essay on the Shaking Palsy,” described Parkinson's disease (PD) as a progressive neurodegenerative disease, characterized by selective loss of dopaminergic neurons in the human midbrain region known as the substantia nigra pars compacta (SNpc) [1]. Degeneration of dopaminergic neurons results in the depletion of the dopamine neurotransmitter production, which manifests clinically as motor dysfunctions such as tremors of hands, bradykinesia, postural instability, and rigidity [2]. Parkinson's disease is also associated with the presence of *α*-synuclein inclusions known as the Lewy bodies in the* substantia nigra* [3]. However, the basis of selective neuronal loss is still elusive since the disease is only diagnosed at the advanced stage.

The etiology of PD is not clearly defined as the disease does not present any clinical symptoms at the early stage [4]. In most instances, by the time the patient experiences the first clinical symptoms, about 50–70% of the dopaminergic neurons have been damaged or degenerated [5]. Up to now, the factors that trigger the onset of this disease still remain unknown [6]. Although, it has been proposed that PD may be caused by a genetic predisposition or environmental toxins, there is no direct evidence to substantiate these claims [6]. However, researchers have delineated the accumulation of abnormal or “toxic” protein in the neuronal cells to be one of the major causes of neuronal death [7]. There is also evidence to support the role of oxidative stress and imbalance in the natural antioxidant defense system, which could be contributing factors that support the formation and/or accumulation of abnormal or toxic proteins in the neurons [8]. Other factors associated with the pathogenesis of PD include living in rural areas [9], farming activity [10], and drinking well-water [11], as these factors can cause exposure to neurotoxic agents that are usually found in pesticides and environmental toxins [12]. On the contrary, some factors like regular consumption of caffeine and tea [13] as well as smoking [14] have been found to exert some protective effects against the onset of PD. A few prospective cohort studies as well as animal studies have suggested and proven that moderate to vigorous physical exercise can be a prophylaxis measure against PD [15, 16].

Flavonoids are water-soluble, broad polyphenol family found ubiquitously in plants, which contribute to the orange, blue, and purple color of fruits, flowers, and leaves [17]. Currently, more than 8000 flavonoid compounds have been identified and these are distributed in various kinds of food such as fruits, grains, nuts, green and black tea, and vegetables [18]. Primarily these flavonoids are synthesized by plants via the photosynthesis process and functions in protecting plants against reactive oxygen species (ROS) and are consumed by herbivores [19]. As shown in Table 1, flavonoids can be classified into six main subgroups: flavonones, flavones, isoflavones, flavanols, flavanones, and anthocyanidins [20].

Within the last 20 years, there has been an explosion of facts on the protective effects of flavonoids. However, the initial information on the health benefits of flavonoids relates to the profound antioxidant properties of these compounds [21]. To date, there is growing evidence that flavonoids do not only exhibit antioxidant effects, but also exhibit a variety of other protective effects such as antiapoptotic [22], anticancer [23], anti-inflammatory [24], antiviral [25], and antibacterial [26] effects. Interestingly, flavonoid compounds benefited humans in overcoming oxidative damage-related diseases such as cancer, atherosclerosis, asthma, neurodegenerative disease like PD and Alzheimer's disease (AD) [27]. In this review, the current literature on the protective mechanisms of flavonoids in delaying neuronal cell loss in Parkinson's disease is discussed in depth.

## 2. Activation of Intracellular Antioxidant Enzymes

Several studies have suggested that PD is a consequence of free radicals-induced oxidative stress [28–30]. In a normal state, free radicals are usually detoxified by various internal antioxidant enzymes to less toxic molecules, which are then removed by various ways [31]. However, the natural antioxidant defense systems may not be able to support overwhelming production of free radicals and this could result in a reduction in the activity of these enzymes [23]. Hence, increased free radicals and oxidative stress in cells can culminate in damaging biological molecules including DNA, proteins, and carbohydrates and cell death [32]. The antioxidant enzymes are including superoxide dismutase (SOD), glutathione peroxidase (GPx), and catalase (CAT) that facilitate reactions that help to catalyze the ROS to less toxic molecules [33] (Table 2), thereby playing a key role in preventing lipid peroxidation [23, 24].

Flavonoid compounds have been found to activate the endogenous antioxidant status in neuronal cells hence protecting them from undergoing neurodegeneration [31]. Polyphenols such as quercetin glycosides, rutin, and isoquercitrin (Table 3) have distinct features in upregulating the production of intracellular antioxidant enzymes such as SOD, GPx, CAT, and glutathione in a 6-hydroxydopamine- (6-OHDA-) induced in PC-12, rat pheochromocytoma cells [31, 34, 35]. Besides that, quercetin, fisetin, methyl gallate, and propyl gallate were also found to protect neuronal cells from oxidative stress through elevation of intracellular glutathione level [20]. Apart from that, the neuroprotective effects of polyphenols in protecting neuronal cells were further demonstrated in animal studies using naringin [36]. In this study, naringin was found to suppress the 3-nitropropionic acid-induced neuronal apoptosis via activation of SOD, GPX, CAT, and GR (glutathione reductase) in both striatum and plasma of Wistar rats [36]. Genistein [37] and naringenin [38] also have proved to elevate the antioxidant enzymes, namely, superoxide dismutase, and glutathione peroxidase. Although there are numerous findings on the activation of antioxidant enzymes by flavonoids polyphenols, the mechanisms of action is still unclear.

Several studies have investigated the correlation between free radicals and antioxidant enzymes and a vast number of findings have shown increase in free radical-induced oxidative damage in* in vitro *test systems to be indirectly proportional to the activation of internal antioxidant enzymes [31]. Intriguingly, flavonoids caused activation of these antioxidant enzymes in free radical-induced test systems [31]. Along with this, the activation of intracellular enzymes can be explained by two mechanisms; (i) flavonoids attenuated the free radicals-induced damage on antioxidant enzymes by scavenging the radicals [51] and (ii) flavonoids bound with the antioxidant enzymes and caused direct activation of these enzymes, where any of these mechanisms will result in increased activity of the antioxidant enzyme [31]. In one of the earlier discoveries, Nagata and coworkers (1999) have shown that the antioxidant effects of quercetin and catechin are mediated by direct interaction with the GPx enzyme [52]. These flavonoids cause modulation in the structure-activity of GPx and thereby enhanced its antioxidant activity [52, 53]. In a similar study, it was found that addition of rutin, quercitrin, myricetin, and kaempferol to catalase had a direct effect of activation of catalase and this effect was attributed to the binding of these polyphenols to heme moiety or protein region of this enzyme [54].

## 3. Suppression of Lipid Peroxidation in Parkinson's Disease

Free radicals and ROS are generated as by-products of several normal cellular functions such as the mitochondrial oxidative phosphorylation system, phagocytosis, and the arachidonic acid metabolism pathway [55]. Most ROS such as the superoxide anion (O_2_^•^), hydrogen peroxide (H_2_O_2_), nitrogen species (NO), hydroxyl (OH^−^), and alkoxyl radicals are hazardous to cells until it is well catabolized to its less toxic substance by the natural antioxidant enzyme systems. However, if the consistent build-up of ROS and free radicals cannot be supported by the various antioxidant enzyme systems, these conditions can result in oxidative stress and lipid peroxidation, which eventually lead to cellular damage. Several studies have suggested that the presence of neurotoxic substances in the human brain may augment the ROS-induced oxidative damage [56–58]. For instance, in PD, prolonged exposure to neurotoxins such as paraquat and 1-methyl-4-phenyl-1,2,3,6-tetrahydropyridine (MPTP) leads to increased generation of ROS in brain neurons as these toxic substances could not be effectively removed by the natural antioxidant enzymes in the brain. This, in turn, inhibited the mitochondrial complex I system, oxidation of polyunsaturated fatty acid (PUFA), protein aggregation, and DNA damage in the neuronal cells [59].

Pryor and Porter were the pioneers in suggesting that lipid peroxidation of certain polyunsaturated fatty acids (PUFA) produces 4-hydroxy-2-nonenal (HNE) as one of the many by-products [60]. HNE is an interesting by-product as it is cytotoxic and appears to be involved in various degenerative diseases, including diabetes [61], pulmonary diseases [62], and Parkinson's disease [63]. In Parkinson's disease, for instance, HNE is found to be an effective protein modifier that induces cross-linking of the monomeric *α*-synuclein molecules, thereby converting these proteins into high molecular weight *β*-sheet-rich oligomers [63, 64]. The *α*-synuclein is a soluble protein consisting of 140 amino acid molecules and is usually located at the presynaptic regions of neurons. Several studies have suggested that *α*-synuclein is involved in neurotransmitter secretion as well as in the regulation of synaptic vesicle pool and plasticity [65–67]. In the pathogenesis of PD, it has been proposed that oxidative stress triggers a vicious cycle by inducing lipid peroxidation and accumulation of *α*-synuclein aggregates, which forms Lewy bodies, which forms Lewy bodies, which are associated with neuronal dysfunction that triggers the onset of PD symptoms [68, 69].

There is a substantial body of evidence, which suggest that flavonoid-rich cocoa-derived foods possess free radicals scavenging property against the superoxide anions such as H_2_O_2_, HClO, and peroxynitrite [70, 71]. The flavan-3-ol compounds present in cocoa are the monomers catechin and epicatechin and the dimer procyanidin B2. These compounds were shown to inhibit lipid peroxidation in brain homogenates and human plasma via anon-enzymatic system [70, 72]. Inhibition of lipid peroxidation in neuronal cells could help to delay the ongoing neurodegeneration process in PD [73]. Besides that, quercetin glycoside derivatives, rutin, and isoquercitrin have shown potent antioxidant potential by attenuating lipid peroxidation induced by 6-OHDA on PC12 neuronal cells (Table 3) [31, 34, 35]. In addition, black tea extract, which contains epigallocatechin (EGCG) polyphenol, was also reported to suppress lipid peroxidation in a 6-OHDA induced rat model of PD [74, 75]. The black tea pretreated rats showed attenuation of lipid peroxidation by 59% compared to the rats in the control group, which were only treated with 6-OHDA [75]. The polyphenol theaflavin was reported to inhibit xanthine oxidase (XO), an enzyme involved in producing superoxides, hence protecting the neuronal cells from undergoing lipid peroxidation [76]. The common feature of most polyphenols, that is, their antioxidant property, is only evident during oxidative stress condition and is not usually demonstrable under normal condition [75]. These convincing evidences markedly support the ability of flavonoids to exert neuroprotective roles via scavenging ROS generated during oxidative stress and subsequently suppress lipid peroxidation in neuronal cells or in animal models of PD.

## 4. Inhibition of Proinflammatory and Proapoptotic Mediators

**Figure 1:**
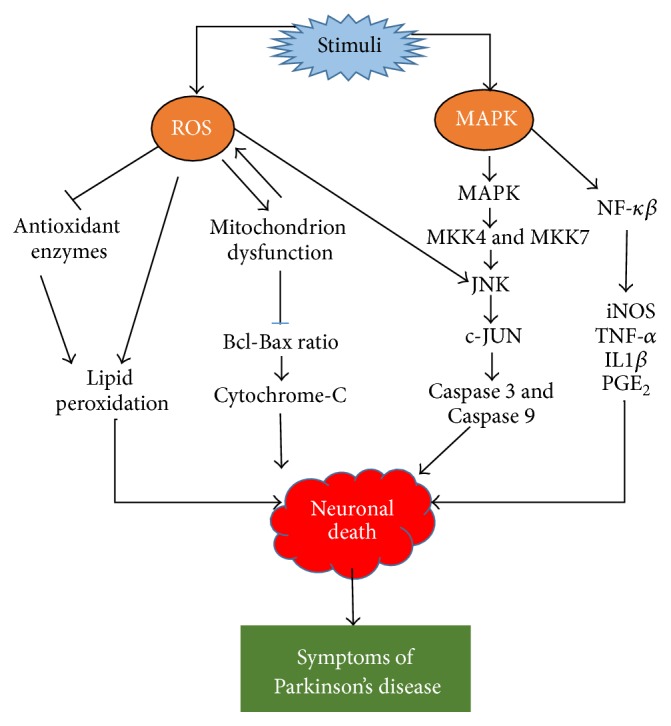
Simplified depiction of ROS and MAPK-induced cytotoxicity. External stimuli including neurotoxin or lipopolysaccharide could generate ROS that is able to suppress the endogenous antioxidant enzymes particularly superoxide dismutase, glutathione peroxidase, and catalase and leads to increase in lipid peroxidation and cell death. The ROS has the ability to directly cause lipid peroxidation and cellular damage as well affecting the mitochondria metabolism, which suppresses the Bcl-Bax ratio and result in leakage of cytochrome-c from mitochondria and eventually cell death. The presence of external stimuli activates MAPK-induced inflammatory mediators including JNK and c-JUN that cause activation of proapoptotic caspases, namely, Caspase 3 and Caspase 9; and the effect is cellular apoptosis. The MAPK family is also responsible in initiating the NF-*κ*B induced expression of proinflammatory cytokine genes (iNOS, TNF-*α*, and ILI*β*). The symptoms of Parkinson's disease occur as a result of neurodegeneration of dopamine producing neurons.

Several lines of evidence suggest that microglia activation has a close association with the pathogenesis of PD [77, 78]. This is in line with the discovery of high levels of microglia activation in the vicinity of degenerating dopaminergic neurons and other areas of the human brain such as hippocampus, cingulate cortex, and temporal cortex [79, 80]. Microglia activation is initiated by the presence of extracellular stimuli including endotoxin, cytokines, misfolded or damaged proteins, and chemokines [81]. In the event of microglial activation, the redox-sensitive nuclear factor-kappa-B (NF-*κ*B) found in the cytoplasm will translocate to the nuclear compartment of the cell and form adducts with the DNA. This results in the activation of various proinflammatory genes such as interleukin-1 beta (IL-1*β*), tumor necrosis factor-alpha (TNF-*α*), cyclooxygenase-2 (COX-2), and inducible nitric oxide synthase (iNOS) as well as IL-6 (Figure 1) [82, 83].

Some studies have found neuroinflammation to be associated with the pathogenesis of PD. Hence, a host of anti-inflammatory therapies have been tested using cell-based and rat models of PD to test the ability of various neuroinflammatory mediators such as Dexamethasone [84], aspirin [85], interleukin-10 [86], and Minocycline [87] to suppress the onset of Parkinson-like symptoms. However, one potential approach to ameliorate the neuroinflammation process is by applying natural polyphenols as the therapeutic agent, since these compounds do not have any significant known adverse effects and they appear to promise almost similar outcomes as conventional drug therapies [88]. The potency of various polyphenols tested using both cell-based and animal model of PD is summarized in Table 3. Various flavonoids such as genistein [89], morin [90], kaempferol [91], and emodin [92] were reported to suppress secretion of TNF-*α*. A recent study found decreased expression of* NF-κB*,* iNOS, *and* COX-2 *genes in naringenin pretreated Wistar rats in an experimental model of focal cerebral ischemia/reperfusion (I/R) induced inflammation [83]. In this study, naringenin upregulated the antioxidant status in the naringenin-treated rats as well as inhibiting the expression of* NF-κB *and activation of downstream genes that can trigger the inflammation cascade [83]. Apigenin has gained particular attention as an anti-inflammatory agent that inhibit the expression of nitric oxide (NO), iNOS, and COX-2 in lipopolysaccharide- (LPS-) induced RAW 264.7 cells [93].

Interestingly, there are also studies that show that flavonol polyphenol myricetin can increase the Bcl/Bax ratio as well as decreasing Caspase 3 expression in 1-methyl-4-phenyl-1,2,3,6-tetrahydropyridine (MPP^+^) induced cell model of PD (Figure 1) [94]. The Bcl-2 family is associated with mitochondrial function and plays a pivotal role in the activation of caspases and apoptosis [95]. The Bcl-2 protein is an antiapoptotic agent that binds to Bax, which decreases the proapoptotic effect of Bax by forming Bcl/Bax heterodimers, a preset ratio that determines the survival or death of cells following an apoptotic stimulus [96]. Hence, an increase in the Bcl/Bax ratio by myricetin pretreatment increased the survival of the neuronal cells [42]. Genistein, an isoflavone, largely found in Soy bean has been shown to possess antiapoptotic effects in 6-OHDA induced SK-N-SH human neuroblastoma cells, where genistein was found to attenuate upregulation of Bax induced by exposure 6-OHDA as well as downregulating the expression of Bcl-2 mRNA and protein [97].

Flavonoids can also exert its protective mechanism via modulation of the mitogen-activated protein kinase (MAPK) signaling pathways [98]. During oxidative stress, the MAPK pathways are activated, ensuing phosphorylation of MAPK kinase 4 (MKK4), a unique protein among the MKK family that is widely distributed in rat and human brains, particularly in the cerebral cortex, hypothalamus, and hippocampus [99]. The stress-induced phosphorylation of the MKK4 results in activation of extracellular signal-regulated kinase 5 (ERK5), c-Jun N-terminal kinase (JNK), and p38 that activates downstream proinflammatory mediators (Figure 1) [100]. Apigenin was found to protect neuronal cells via suppressing the phosphorylation of p38, MAPK, and JNK but not the ERK pathway [93]. A similar finding was observed with theaflavins and thearubigins, the major polyphenols of black tea, whereby a sustained activation of the p38 MAPK and JNK were observed but not in the ERK pathway [101]. Moreover, cocoa procyanidin binds directly to MKK4, inhibiting its activity and also suppressing the JNK signaling pathway [102]. In most of these studies, polyphenols have been reported to attenuate the proinflammatory and proapoptotic mediators and protect the neuronal cells.

## 5. Mitochondria Targeted Flavonoid Therapy

Mitochondria, a defined cytoplasmic organelle, plays a pivotal in cellular aerobic respiration and regulation of Ca^2+^ homeostasis and is also involved in orchestration of cellular apoptosis and production of ROS [103, 104]. Dysfunctions in the physiological processes of mitochondria can lead to the onset of age-related diseases such as PD and AD [104]. In line with recent understanding, mitochondrial dysfunction can be caused by deficiency in Complex I, which plays a crucial role in mitochondria respiration chain [105]. Exposure of neuronal cells to neurotoxins such as MPP^+^, 6-OHDA, and paraquat causes selective uptake of these toxins by the dopaminergic neurons where these toxins inhibit the activity of Complex I [106]. The decrease in Complex I activity produces excess superoxide radicals that are capable of overwhelming the natural antioxidant systems and eventually cause oxidative stress and neurodegeneration [107]. Several studies have shown mitochondrial ROS production in cells to be the most important source of ROS despite other sources such as nicotinamide adenine dinucleotide phosphate (NADPH) oxidase (NOX) [108], XO, cytochrome P450, and the mitochondrial electron transport chain (ETC) [109]. The main reason for mitochondrial ROS to garner mounting attention is its ability to directly activate mediators of proinflammatory cytokine and MAPK [110], which can lead to several pathological conditions such as cancers, cardiovascular, and neurodegenerative diseases.

Mitochondrial dysfunction can also be caused by the accumulation and aggregation of amyloid-beta (A*β*) peptides [111]. Occurrence of A*β* peptides in neuronal cell is a pathological hallmark in neurodegenerative diseases, particularly PD and AD [112]. A recent study has proposed that soluble amyloid aggregates that are formed in neuronal cells have the inherent capacity to penetrate the mitochondrial membrane and induce neuronal death [113]. The penetration of the A*β* peptides occurs through the mitochondria-associated endoplasmic reticulum membranes, which is a physical connection between the membrane of the endoplasmic reticulum and the mitochondrial outer membrane [113]. Hence, mitochondria targeted polyphenol therapy is an excellent approach in modulating mitochondrial dynamic, function, and biogenesis [114].

Quercetin polyphenol is one of the flavonoids compounds that is widely investigated and reported for its antioxidant [115], anticancer [116], anti-inflammatory [117], and antiviral [118] effects. Interestingly, a recent study has shown that quercetin has the ability to repair the mitochondrial electron transport defect in a rotenone-induced rat model of Parkinsonism (Table 2) [43]. This study also demonstrated a dose-dependent upregulation of Complex I activity in the mitochondria, which the authors attribute to the powerful hydroxyl radicals scavenging action of quercetin [43]. In another study, EGCG, a natural polyphenol derived from green tea, was reported to restore mitochondrial energy deficit in lymphoblasts and fibroblasts from Down syndrome patients [119]. The protective mechanism of EGCG is not clearly defined, but it is proposed that Complex I activity and ATP synthase catalytic activities have been activated beside promotion of cellular levels of cyclic adenosine monophosphate (cAMP) and protein kinase A (PKA) dependent phosphorylation of Complex I. Treatment with EGCG effectively stimulated mitochondrial biogenesis in the lymphoblasts and fibroblasts Down syndrome patients via activation of the Sirtuin 1 (SIRT1) dependent Peroxisome proliferator-activated receptor-*γ* coactivator (PGC-1*α*), nuclear respiratory factor-1 (NRF-1), and mitochondrial DNA content [119]. There is also accumulating evidence that supports the protective effect of genistein on neuronal cells against oxidative damage [120] and glutamate and A*β* amyloid toxicity [121]. Furthermore, it has been reported that genistein exerts its protective mechanism via restoring mitochondrial membrane potential that was significantly decreased by 6-OHDA treatment in SK-N-SH neuroblastoma cells [97]. Naringin, a ubiquitously found flavanone glycoside, has been reported to exhibit several protective effects including antioxidant, ROS scavenging, and metal chelating activities [122–124]. Besides improving cognitive dysfunction and oxidative defense, it was reported that naringin can restore mitochondrial enzyme functions, specifically Complexes I and III activity in a murine model [38].

## 6. Modulation of Gene Expression Changes

Neuronal cells that undergo a programmed cell death or apoptosis are regulated by both “*protective*” and “*destructive*” genes. “*Protective genes” *are genes that execute protective mechanism by suppressing oxidative stress, thereby protecting cells, such as thioredoxin reductase-1, glutathione S-transferase, pi 2 (Gstp2), superoxide dismutase (SOD2), copper chaperone for SOD1 (CCS), glucose-6-phosphate dehydrogenase (G6PD), and Bcl-2 [125]. In contrast, increased expression of certain genes such as* neuronal cell death-inducible putative kinase* (NIPK),* ankyrin repeat domain-3 (Ankrd3)*,* protein phosphatase 1G (Ppm1g)*, and* ubiquitin carboxyl-terminal hydrolase-20* results in cellular death in PC-12 cells following exposure to 6-OHDA [125]. Studies on PD model showed that treatment of dopaminergic cells (e.g., PC12 cells) with neurotoxins like 6-OHDA or MPTP upregulated proapoptotic genes and other “*destructive*” genes that promote cellular apoptosis [125]. Hence, therapeutic drug targeting these “*destructive*” genes may protect neurons from undergoing apoptotic process and neuronal death.

In our previous study, we have shown that quercetin glycosides, rutin, and isoquercitrin induced neuroprotection by changes in gene expression in 6-OHDA treated PC-12 rat pheochromocytoma cells [126]. Rutin pretreatment attenuated the expression of* Parkin 5 (Park 5)*,* Parkin 7 (Park 7)*,* Caspase 3*,* Caspase 7*, and* Ataxin 2* gene expression that were highly expressed by 6-OHDA. Moreover, rutin upregulated the protective genes, including* tyrosine hydroxylase (Th)*,* neuron specific gene family member 1 (Nsg1), N-ethylmaleimide-sensitive factor (Nsf)*, and* optic atrophy 1 homolog (Opa1)* genes [126]. On the hand, isoquercitrin suppressed* Park 5* and* Park 7* genes and stimulated* Nsf* and* Nsg1* genes [126] A recent study found that quercetin inhibited NO production by suppressing inducible the transcription of the* iNOS *gene [127]. Quercetin reportedly exerted this effect by suppressing the signaling pathway that leads to the activation of NF-*κ*B, activating protein-1 (AP-1) and signal transducer, and activator of transcription-1 (STAT1), which are the key intracellular agents that contribute to the neuroinflammatory process [128]. This study also found that quercetin upregulated the expression of the* heme oxygenase-1 *gene in the BV-2 microglia cells [128]. The heme oxygenase-1 was recently named the “therapeutic funnel” as it was found to possess anti-inflammatory and antiapoptotic effects [129]. In addition, quercetin-induced* heme oxygenase 1* gene expression was reported to be related to the activation of tyrosine kinase and MAPK [127].

Emerging evidence suggests that genistein, an isoflavone naturally present in Soy beans, reduced MPTP-induced neurotoxicity in a murine model of PD via activation of the Bcl-2 mRNA level in the midbrain [130]. Bcl-2 is an antiapoptotic regulatory protein that maintains mitochondrial integrity by inhibiting the release of cytochrome-c and the intrinsic cascade that leads to activation of Caspase 3 and apoptosis [131]. The neuroprotective mechanism of genistein was attributed to its increased affinity towards the estrogen receptor (ER) hence affecting estrogen-regulated* Bcl-2 *gene expression [130]. Previous studies have shown that estradiol treatment could stimulate* Bcl-2* expression in the hypothalamus [132], cerebral cortex [133] as well as neuronal cell line [134]. Therefore, isoflavone genistein could be an effective neuroprotective therapy to reduce the neurotoxin-induced dopaminergic neuronal loss and increase cell survival in human midbrain. Amongst different flavonoids, epigallocatechin-3-gallate (ECGC) was found to modulate changes in gene expression in neuronal cell cultures [135]. Exposure to EGCG was reported to attenuate 6-OHDA induced neuronal loss by preventing the expression of proapoptotic genes such as* Bax*,* Bad, *and* Mdm2 *and decrease the expression of antiapoptotic gene expression including* Bcl-2*,* Bcl-w*, and* Bcl-x(l)* [135].

## 7. Summary

Polyphenol flavonoids are found ubiquitously in a wide range of fruits and vegetables such as apple skin, celery, oranges, onion, mango, apples, and buckwheat, as well as food and beverages derived from plants including olive oil, black/green tea, and red wine. Over the last two decades, a significant amount of data pertaining to the antioxidant effects of different types of flavonoids has been documented. Studies to validate neuroprotective effects of flavonoids were performed induced neurotoxins with either pre- or posttreatment with flavonoid compounds based on the objective of the study. Almost all the published literature suggested that flavonoids can exert neuroprotective effects in pathological conditions, that is, in the presence of prooxidants or neurotoxins but not under normal physiological conditions. These findings clearly explain the antioxidant nature of flavonoids in arresting free radical-induced oxidative damage, which is known to be central to many degenerating diseases including PD. Various types of flavonoid were tested in many types of disease model in both* in vitro *and* in vivo *experimental set-ups. Some of the many protective effects of flavonoids reported included antiapoptosis, antibacterial, antiviral, antioxidant, anticancer, antidiabetic, and anti-inflammatory. However, in terms of neuroprotection, the antiapoptotic and anti-inflammatory ability of flavonoids appear to impede the progressive neuronal loss in neurodegenerative diseases particularly PD. Apart from that, flavonoids such as quercetin, rutin, isoquercitrin, and catechin were found to increase the levels of the natural antioxidant enzymes in the cellular compartment as a bid to suppress the free radical-induced lipid peroxidation. Besides that, flavonoids were also found to downregulate the neuroinflammation process by inhibiting the MAPK signaling pathways that can attenuate the activation of the ERK5, JNK, and p38 signalling pathways, which stimulate production of more downstream proinflammatory mediators. In addition, EGCG was found to modulate expression of proapoptotic genes like* Bax*,* Bad*, and* Mdm2* whilst genistein induced changes in the expression of the* Bcl-2* gene, thereby increasing survival of cells in neurodegenerative diseases. Although flavonoids have shed some light as neuroprotective agents, there are many barrels and barricades in this area of research. To date, the pathogenesis of PD is still poorly defined as the main trigger of the dopaminergic neuronal loss is still largely unknown, although some of the main contributing risk factors like genetic predisposition, environmental toxins, and lack of exercise have been identified. Elucidation of the pathogenesis of PD will further aid in the search to identify flavonoid compounds to stop the trigger point of the “domino” cascade of events involved in neuronal cell death.

## 8. Future Perspectives

The design of the “magic bullet” as a therapeutic approach to help either prevent or treat PD depends on our understanding of the mechanisms by which flavonoids counteract neuronal damage. Although some mechanisms have been described well, we are still far from getting the complete picture of protective mechanism of flavonoid polyphenol. There are many loop holes in the comprehension of the mechanism by which flavonoids protect neuronal cell; for instance, (i) studies evaluating flavonoids to promote neuronal function and neurite outgrowth in human dopaminergic neurons are limited; (ii) clinical trials of neuroprotection evidence of most promising flavonoid polyphenols in PD patient are scarce; and (iii) strategies to introduce flavonoids and therapeutic dosage as these molecules change compositions in* in vivo *system upon exposure to the acidic environment of gastric cavity and finally studies evaluating the ability of emerging flavonoids compounds to cross the blood brain barrier are needed as very few flavonoids have been tested for this ability in animal models.

## Figures and Tables

**Table 1:** 

Subgroups	Types of flavonoids	Structures	Food sources
Flavone	Apigenin Luteolin	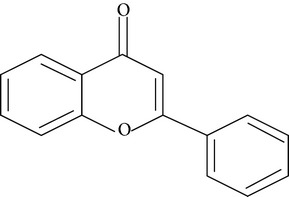	Apple skins Celery

Flavonol	Kaempferol Myricetin Quercetin Quercetin glycosides, rutin Quercetin glycosides, isoquercitrin	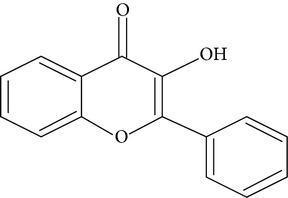	Broccoli Fruits peels Lettuce, olives, onions Buckwheat, citrus fruits Mango, apples, onion

Flavanol	(−)−Epicatechin (−)−Epicatechin 3-gallate (−)−Epigallocatechin (−)−Epigallocatechin 3-gallate (+)−Gallocatechin	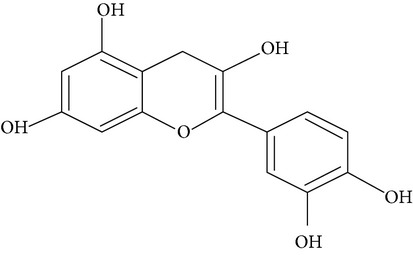	Berries, blueberries, fava beans, mature seeds, broccoli, Brussels sprouts

Flavanone	Hesperetin Fisetin Naringin Naringenin	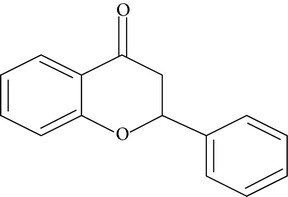	Citrus peel Citrus fruit

Anthocyanidin	Cyanidin Delphinidin Malvidin Pelargonidin Petunidin	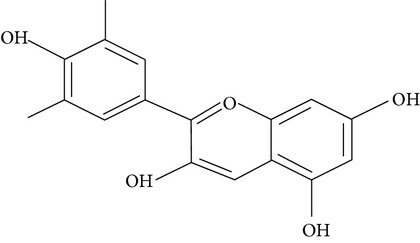	Berries Cherries Grapes Raspberries Red wines, strawberries, tea

Isoflavone	Daidzein Genistein Glycitein	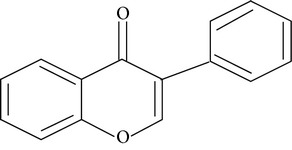	Soy bean

**Table 2:** 

Antioxidant enzyme	Function	Chemical reaction

Superoxide dismutase	Catalysing superoxide anion to oxygen and hydrogen peroxide	2O_2_^•^ + 2H^+^ → H_2_O_2_ + O_2_

Catalase	Detoxifying hydrogen peroxide to water and oxygen molecule	2H_2_O_2_ → O_2_ + 2H_2_O

Glutathione (GSH)	Electron donor to GPx in reducing hydroperoxides to water molecules	2GSH + H_2_O_2_ → GS–SG + 2H_2_O

Glutathione peroxidase	Reducing hydroperoxides to water molecules	2GSH + H_2_O_2_→ GS–SG + 2H_2_O

Glutathione reductase	Catalyzing the reduction of glutathione disulfide (GSSG) to the sulfhydryl form glutathione (GSH)	GSSG + NADPH + H^+^ → 2GSH + NADP^+^

**O**_2_^•^ (superoxide anion);  **H**_2_ **O**_2_ (hydrogen peroxide);  **O**_2_ (oxygen);  **H**_2_ **O** (water molecule);**  GSSG** (reduced glutathione); **NADPH** (nicotinamide adenine dinucleotide phosphate).

**Table 3:** 

Types of polyphenol	Studied model: cell or animal	Outcome	References
Apigenin	BV-2 murine microglia cell line and cerebral artery occlusion-induced focal ischemia in mice	(i) Inhibiting production of nitric oxide and prostaglandin E2 (ii) Suppressing p38 mitogen-activated protein kinase (MAPK), c-Jun N-terminal kinase (JNK) phosphorylation (iii) Protecting neuronal cells from injury in middle cerebral artery occlusion	Ha et al., 2008 [39]

Luteolin	Lipopolysaccharide (LPS) induced primary mesencephalic neuron-glia	(i) Attenuating the decrease in dopamine uptake and loss of tyrosine hydroxylase (ii) Inhibiting activation of microglia and excessive production of tumor necrosis factor-*α*, nitric oxide, and superoxide	Chen et al., 2008 [40]

Kaempferol	Rotenone-induced SH-SY5Y cells and primary neurons	(i) Enhancing mitochondrial turnover by autophagy	Filomeni et al., 2012 [41]

Myricetin	MPP^+^-treated MES23.5 cells	(i) Attenuating cell loss and nuclear condensation (ii) Suppressing the production of intracellular reactive oxygen species (ROS) (iii) Restoring the mitochondrial transmembrane potential (iv) Increasing Bcl-2/Bax ratio and decreasing Caspase 3 activation (v) Decreasing phosphorylation of MAPK kinase 4 and JNK	Zhang et al., 2011 [42]

Quercetin	Rotenone-induced rats	(i) Reducing cell loss in striatal dopamine (ii) Scavenging hydroxyl radicals (iii) Upregulating mitochondrial complex-I activity	Karuppagounder et al., 2013 [43]

Rutin	6-OHDA induced PC-12 neuronal cells	(i) Activating antioxidant enzymes (SOD, CAT, GPx, GSH) (ii) Suppressing lipid peroxidation	Magalingam et al., 2013 [31]

Isoquercitrin	6-OHDA induced PC-12 neuronal cells	(i) Activating antioxidant enzymes (SOD, CAT, GPx, GSH) (ii) Suppressing lipid peroxidation	Magalingam et al., 2014 [34]

Catechin	6-OHDA-lesioned rats	(i) Attenuating the increase in rotational behavior (ii) Improving the locomotor activity (iii) Restoring GSH levels, increasing dopamine and DOPAC content	Teixeira et al., 2013 [44]

(−)−Epigallocatechin 3-gallate	Serum deprived human SH-SY5Y neuroblastoma cells	(i) Inducing the levels of beta tubulin IV and tropomyosin 3 (ii) Increasing the levels of the binding protein 14-3-3 gamma (iii) Decreasing protein levels and mRNA expression of the beta subunit of the enzyme prolyl 4-hydroxylase (iv) Decreasing protein levels of the immunoglobulin-heavy-chain binding protein and the heat shock protein 90 beta	Weinreb et al., 2007 [45]

Hesperidin	6-OHDA induced aged mice	(i) Preventing memory impairment (ii) Attenuating reduction in GPx and CAT activity, total reactive antioxidant potential, and the DA and its metabolite levels in the striatum (iii) Attenuating reactive species levels and glutathione reductase	Antunes et al., 2014 [46]

Fisetin	lipopolysaccharide (LPS) stimulated BV-2 microglia cells	(i) Suppressing the production of TNF-*α*, nitric oxide, and PG E_2_ (ii) Inhibiting the gene expression of TNF-*α*, interleukin (IL-1*β*), COX-2, and (iNOS) at both mRNA and protein levels. (iii) Suppressing I*κ*B degradation, nuclear translocation of NF-*κ*B, and phosphorylation of p38 MAPKs	Zheng et al., 2008 [47]

Naringenin	6-OHDA induced SH-SY5Y cells and mice	(i) Increasing in nuclear factor E2-related factor 2 (Nrf2) protein levels and activating of antioxidant response pathway genes (ii) Protecting nigrostriatal dopaminergic neurons against neurodegeneration and oxidative damage	Lou et al., 2014 [48]

Theaflavin	MPTP-induced mouse	(i) Reducing oxidative stress (ii) Improving motor behavior and expression of dopamine transporter and vesicular monoamine transporter 2 in striatum and substantia nigra.	Anandhan et al., 2012 [49]

Proanthocyanidin	Rotenone in a primary neuronal cell	(i) Protecting dopaminergic cell (ii) Rescuing mitochondrial respiration in a dopaminergic cell line	Strathearn et al., 2014 [50]
